# Role of regular preventive dental visits in moderating lifestyle-related disease medical expenditures among employed adults

**DOI:** 10.1371/journal.pone.0358693

**Published:** 2026-10-15

**Authors:** Wakana Oyoshi, Terumitsu Murakami, Hiraki Tsushima, Kazue Kawamoto, Akinori Igarashi, Shintaro Yamaoka, Hiroshi Fukuda

**Affiliations:** 1 Digital Strategy Department, Lion Corporation, Tokyo, Japan; 2 Human Resources Development Center, Lion Corporation, Tokyo, Japan; 3 Department of Advanced Preventive Medicine and Health Literacy, Graduate School of Medicine, Juntendo University, Tokyo, Japan; University of Southampton, MALAYSIA

## Abstract

This study aimed to elucidate the impact of preventive dental visits (PDVs) over a 9-year follow-up period on cumulative medical expenditures (MEs) related to lifestyle-related diseases. To this end, the study included 1,891 full-time employees (1,478 males and 413 females), aged 19–57 years, of a manufacturing company in Japan over a 9-year period. This historical cohort study was conducted from 2015 to 2023. The year 2015 served as the baseline, with the subsequent 8 years (2016–2023) used for follow-up. Based on an 8-year dental check-up follow-up survey from 2016 to 2023, participants were classified into two groups, those who reported PDVs in ≥87.5% of the years they answered the survey (regular PDVs) and those who reported PDVs in <12.5% of those years (irregular PDVs). Using 2015 as the baseline, adjustments were made for sex, age, division, Community Periodontal Index, oral health literacy, number of outpatient visits, annual MEs, annual lifestyle-related diseases medical expenditures (LDMEs), lifestyle habits, and health risks. Cumulative LDMEs per capita from 2016 onwards were examined in each group classified as with or without regular PDVs. To adjust for confounding variables between the two groups, we performed a propensity score matching. Although no significant differences in the proportion of individuals incurring LDMEs between the groups with and without regular PDVs, the LDMEs per capita among those who incurred expenditures were significantly lower in the group with regular PDVs. In sensitivity analysis, the threshold for group classification was changed. Specifically, the analysis was conducted with participants who had PDVs in ≥50% vs. those who had PDVs in <50% yielded similar findings as those obtained in the main model. Our results suggest that regular PDVs are associated with moderating LDMEs. These findings may serve as a motivation for promoting preventive dentistry in both corporate and public health initiatives.

## Introduction

In recent years, moderating healthcare expenditure has become an urgent policy concern. Health-associated costs have risen steadily in developed countries. In 2023, U.S. healthcare expenditures reached USD 4.87 trillion, marking the largest annual increase since 1990 [1]. Furthermore, Japan’s healthcare spending, recorded at USD 302 billion (exchange rate: JPY 150 per USD; 8.0% of gross domestic product (GDP)) in 2018, is projected to reach USD 522 billion (10.1% of GDP) by 2040 [2]. While various factors contribute to the increase in healthcare expenditure, one significant factor is the rise in lifestyle-related diseases. For instance, a U.S. survey of more than 400,000 individuals showed that the prevalence of type 2 diabetes increased from 10.18% in 2012 to 12.10% in 2022 [3]. Lifestyle-related diseases, such as hypertension, diabetes, and dyslipidemia, which require long-term management, impose a significant burden on healthcare systems. Consequently, moderating lifestyle-related disease medical expenditures (LDMEs) through prevention and management has become a key public health priority. Japan operates a universal healthcare system with mandatory health insurance coverage, where medical costs are shared between patients, employers, and the government. As healthcare expenditures rise, the financial burden on both the insurance system and government increases proportionally. This financial structure makes the moderation of medical expenditures (MEs), particularly those related to preventable conditions such as lifestyle-related diseases, not only a public health concern but also an economic imperative.

Recently, preventive approaches to oral care have gained increasing attention. In 2021, the World Health Organization adopted a resolution on oral health at the 74th World Health Assembly [4]. This resolution promotes a shift from traditional therapeutic care to prevention, including oral health promotion in families, schools, and workplaces. The rationale stems from the fact that many oral diseases are both common and preventable, with several linked to systemic conditions, such as cardiovascular diseases and diabetes. Preventive dental visits (PDVs) alongside self-care are therefore essential. In Sweden, where preventive oral care is strongly emphasized, dental care is fully subsidized until the age of 20 years, after which a fixed government allowance offsets associated costs [5]. In Japan, while dental care is covered under the same universal health insurance system as general medical care, notable differences in preventive care accessibility exist. Preventive dental services, including routine check-ups and cleanings, are primarily paid out-of-pocket, whereas treatment related dental visits are covered by insurance. Furthermore, except for certain occupations, workplace dental check-ups are not legally mandated for adults. This creates a gap in preventive dental care access during working age, despite mandatory dental check-ups provided during school years and for older adults aged 75 and above. Consequently, many employed adults in Japan have limited opportunities for regular dental check-ups unless they proactively seek preventive care at their own expense.

Given these trends, both the moderation of LDMEs and regular PDVs have attracted growing interest. Furthermore, the interrelationship between oral and overall health highlights the need for integrated medical and dental care. Previous studies have examined the relationship between dental care and medical expenditures [6–9], suggesting potential associations between preventive dental services and reduced healthcare costs. However, the specific relationship between regular PDVs and LDMEs remains unclear, particularly over extended observation periods. To address this gap, our study was focused specifically on lifestyle-related diseases because numerous studies have reported associations between oral health and cardiovascular diseases, diabetes, and other lifestyle-related diseases, as highlighted in the World Health Organization’s recommendation for preventive approaches [10, 11]. Because lifestyle-related diseases are influenced by various confounding factors, including age and other lifestyle habits, these factors need to be carefully considered.

Given these points, the objective of this study was to examine the association between regular PDVs and LDMEs while accounting for these potential confounding variables. To this end, we conducted a 9-year historical cohort study and employed propensity score matching to adjust for confounding factors. By investigating the relationship between PDVs and LDMEs, we aimed to contribute evidence that could inform strategies for implementing preventive approaches in oral healthcare.

PDVs were defined as regular dental check-ups and periodic dental scaling (calculus removal). In clinical practice, these preventive visits typically encompass professional tooth polishing, plaque removal, and oral health instruction. These visits are distinct from treatment-related dental visits for symptomatic conditions. Although some companies in Japan voluntarily offer workplace dental check-ups, resource constraints make it difficult to implement such programs across all companies. Therefore, this study focused on PDVs, which are accessible to all working adults in Japan, regardless of whether their employer provides workplace dental check-ups. Understanding the potential benefits of PDVs in moderating LDMEs could encourage voluntary participation in preventive dental care among employed adults.

## Materials and methods

This study was approved by the Institutional Review Board of Lion Corporation (Approval Number: 391). All participants provided written informed consent.

The study enrolled 3,999 employees from 12 business sites of a manufacturing company headquartered in Tokyo, Japan. Data were collected from 2015 to 2023, including medical check-up results, workplace dental check-up results, and receipt data (hereafter collectively referred to as “related data types”). As medical check-ups are legally mandated in Japan, nearly all participants underwent these check-ups. Among the initial 3,999 employees, 2,456 individuals (approximately 61.4%) were continuously eligible from 2015 to 2023 without late participation or dropout. Late participation was defined as the first appearance of related data types in or after 2016 and dropout was defined as the absence of relevant data types in 2023. Even if employees did not have relevant data types in any year from 2016 to 2022 for reasons such as leave of absence, they were included in the analysis if relevant data types appeared in both 2015 and 2023. Participants with missing data were excluded from the analysis (n = 565). Furthermore, to protect participant anonymity and prevent individual identification, individuals whose physical characteristics (height, weight, BMI, or waist circumference) fell outside ±3 standard deviations from the mean (stratified by medical check-up year and sex) were excluded from the analysis.

A total of 1,891 participants whose physical characteristics fell within the required range, provided consent, were enrolled during the study period, and had no missing required data were included in this study. The average age of participants was 41.8 ± 9.4 years, and 78% were male.

Using the questionnaire from dental check-up results for the 8 years from 2016 to 2023, participants were classified into two groups: those with regular PDVs (n = 289), who responded that they had PDVs ≥ 87.5% in the investigated period, and those with irregular PDVs (n = 392) who responded that they had PDVs < 12.5% in the investigated period (Fig 1). This intentional selection of extreme comparison groups was designed to enhance statistical power by comparing individuals with consistently high versus consistently low PDV behavior. PDVs were operationalized as a binary indicator of whether participants reported having at least one PDV in each year (YES/NO). This approach captures individuals who maintain annual engagement with preventive dental services, distinguishing them from those who do not maintain this habit. At the investigated workplace, annual dental check-ups are conducted as part of occupational health programs, during which employees are asked whether they had PDVs in the past year. Participants who did not attend these annual workplace dental check-ups were unable to answer the PDV question for that year. Therefore, classification into regular or irregular PDV groups was based on the percentage of years with reported PDVs relative to the total number of years in which each participant answered the questionnaire, rather than the absolute number of years with PDVs during the 8-year study period. Specifically, if grouping had been performed based on the absolute number of years with PDVs during the 8-year study period, participants who were unable to attend workplace dental screenings owing to reasons such as leave of absence incorrectly classified into the irregular PDV group. This selection bias was eliminated using the percentage-based approach. The distribution of the number of years in which participants answered the dental questionnaire is presented in Table 1. Consequently, participants were classified into the regular PDV group if they reported having PDVs in ≥87.5% of the years in which they answered the questionnaire, and into the irregular PDV group if they reported having PDVs in <12.5% of the years in which they answered the questionnaire.

**Table 1:** Distribution of the number of years in which participants answered the dental questionnaire regarding PDVs.

Number of years in which the questionnaire was answered(during 8 years)	Number of Participants
8	704
7	719
6	301
5	100
4	42
3	12
2	8
1	5
Total	1,891

Note: This table shows the number of years in which each participant answered the questionnaire about PDV attendance during workplace dental check-ups. Participants were classified into regular or irregular PDV groups based on the percentage of affirmative responses (YES to having PDVs) relative to their total number of questionnaire responses, not based on the absolute number of years with PDVs out of 8 years. For example, a participant who answered the questionnaire in 8 years and reported PDVs in 7 of those years (≥7 out of 8 = 87.5%) would be classified in the regular PDV group, as would a participant who answered in 4 years and reported PDVs in all 4 years (4 out of 4 = 100%), because both meet the ≥ 87.5% threshold. Similarly, participants with <12.5% rates (e.g., < 1 out of 8, or 0 out of 4) were classified in the irregular PDV group. Abbreviation: PDV, preventive dental visit

**Fig 1:**
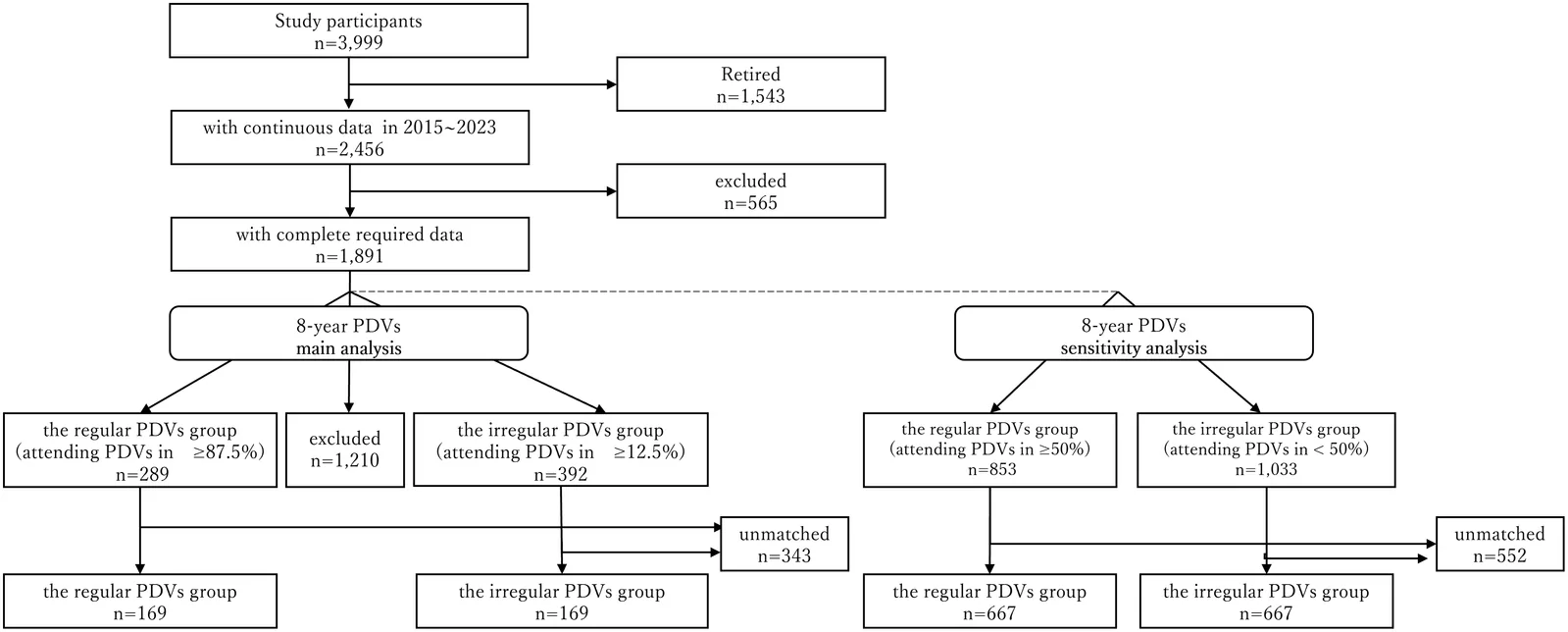
Flow diagram of study participant enrollment. PDVs, preventive dental visits.

For sensitivity analysis, participants were reclassified using a different threshold: those with regular PDVs (n = 858) who responded that they had PDVs in ≥50% of the period, and those with irregular PDVs (n = 1,033) who responded that they had PDVs in <50% of the period. This alternative classification aimed to assess the robustness of the primary findings.

The analysis utilized three datasets covering fiscal years 2015–2023: employee medical expenditure data obtained from the health insurance association to which the employees belonged, medical and dental check-up results provided by the company’s internal health management department. The data were accessed on May 9, 2025. It was necessary to ensure we could not identify individuals from the data. Therefore, the data were processed by the health management department into a form in which individuals could not be identified. The baseline year was set as 2015, and the years 2016–2023 were the observation period.

### Confounding variables

The following data from the baseline year (2015), were used: sex (male/female), age, work division (office work/sales/research/manufacturing/technical/other), the Community Periodontal Index (CPI; probing pocket depth (PPD) ≥4 mm) [12], oral health literacy (whether they remembered last year’s dental check-up results), number of outpatient visits for 1 year (operationally defined based on insurance receipt data as days with insurance claims for medical services excluding those with hospitalization meal coverage, which served as a proxy for outpatient visit days), cumulative MEs for 1 year, and cumulative LDMEs for 1 year. Data were also collected on smoking habits (smoke/quit/do not smoke), exercise habits (hardly ever/one-two times a month/ one-two times a week/three or more times a week), obesity risk (body mass index [BMI] ≥ 25 kg/m^2^ or waist circumference exceeding the following criteria: male ≥85 cm, female ≥90 cm), underweight risk (BMI < 18.5 kg/m^2^), blood pressure risk (systolic blood pressure ≥130 mmHg or diastolic blood pressure ≥85 mmHg), blood glucose risk (fasting blood sugar ≥100 mg/dL or glycated hemoglobin [HbA1c] ≥5.6%), lipid risk (triglycerides ≥150 mg/dL or high-density lipoprotein <40 mg/dL), and liver function risk (meeting one or more of the following: aspartate aminotransferase ≥31 U/L, alanine aminotransferase ≥31 U/L, and gamma-glutamyl transferase ≥51 U/L). Health data, including anthropometric measurements (height, weight, and waist circumference), blood pressure, and blood test results (blood glucose, HbA1c, lipids, and liver function), were collected by health examination staff during the health check-ups. Work division and lifestyle habits, including smoking and exercise habits, and recall of dental check-up results were self-reported by participants through standardized questionnaires administered during the health or dental check-ups. In this study, oral health literacy was operationalized as whether participants remembered their previous year’s dental check-up results. While this proxy measure does not fully capture the comprehensive definition of health literacy—“the cognitive and social skills which determine the motivation and ability of individuals to gain access to, understand and use information in ways which promote and maintain good health” (Nutbeam, 1998) [13]—it partially reflects the ability to retain and recall health-related information, which is one component of health literacy. To ensure the reliability of CPI measurements, systematic calibration was implemented for approximately 20 dental hygienists responsible for the assessments. Specifically, three dental hygienists formed a team, and each independently recorded the CPI code and presence of bleeding as measured on six sites per tooth for the same subject. This procedure was performed twice. For sites where discrepancies were observed among the three examiners’ measurements, a gold-standard dental hygienist who had received training in periodontics as a certified periodontist re-measured the relevant sites, and consensus on appropriate measurement values was reached through discussion. This series of calibration procedures was conducted annually to maintain measurement accuracy and minimize inter-examiner error. Participants exceeding the standard values were considered at risk.

### Research outcomes

The research outcomes were LDMEs, and severe diseases obtained from the receipt data (hereinafter referred to as LDMEs). While the International Classification of Diseases, 10th Revision (ICD-10), is a common global method for classifying medical expenditure data, Japanese claims statistics use a classification method called “122 classifications” (known as “119 classifications” before 2016), which were used in medical benefit surveys [14] and data from the Japan Health Insurance Association [15]. The data used in this analysis was also in a format based on the “122 classifications.” Therefore, to determine whether MEs corresponded to the relevant diseases, ICD-10 codes were mapped to “122 classifications” using a correspondence table, after which LDMEs data were identified. The correspondence table used was the “Social Insurance Tabulation Disease Classification” published by the Ministry of Health, Labour and Welfare of Japan [16]. An excerpt of disease names, ICD-10 codes, and “122 classifications” codes used in this study is shown in Table 2.

**Table 2:** Correspondence between disease names and codes.

ICD-10	Code	122 Classifications	Code
Diabetes	E11-E14	Diabetes	402
Essential Hypertension	I10	Hypertensive Diseases	901
Hyperlipidemia	E78	Lipid Disorders	403
Angina Pectoris	I20	Ischemic Heart Diseases	902
Acute Myocardial Infarction	I21	Ischemic Heart Diseases	902
Intracerebral Hemorrhage	I61	Intracerebral Hemorrhage	905
Cerebral Infarction	I63	Cerebral Infarction	906
Chronic Renal Failure	N18	Renal Failure	1402
Diabetic Nephropathy	E112	Diabetes	402
Diabetic Retinopathy	E113	Diabetes	402

Abbreviations: ICD, International Classification of Diseases

The determination of lifestyle-related diseases was based on the learning materials of the Ministry of Health, Labour and Welfare of Japan [17]. Additionally, while one record in the claims data contains one medical expenditure and multiple diseases, dividing MEs into units smaller than the record level is not possible. Therefore, we focused only on the diseases listed in the “Diagnostic category code 1,” which records the main disease, and extracted those corresponding to specific Diagnostic category codes. MEs corresponding to the disease codes shown in Table 2 were extracted and defined as LDMEs, and the cumulative LDMEs over 8 years were calculated. However, this did not include expenditures on drugs that should be associated with the target diseases. Additionally, for each year, individuals who had health check-up results but no claims data were considered to have not visited a hospital during that year, and their LDMEs were set to USD 0.

### Definition of medical expenditures

All ME data used in this study were derived from receipts data and represent total medical costs comprising both insurer-covered costs and patient co-payments for both inpatient and outpatient services. This definition applies to: baseline MEs, baseline LDMEs as confounding variables, and cumulative LDMEs, which served as the primary research outcome in this study.

### Statistical analysis

As LDMEs do not follow a normal distribution (including Poisson distribution and gamma distributions) with many people having no MEs over 8 years and only some incurring high expenditures, we assessed differences in distribution between the regular and irregular PDV groups using a two-step approach. First, Fisher’s exact probability test was used to confirm whether any difference existed in the proportion of people who incurred MEs between the two groups during the study periods. Second, we used the Mann–Whitney U test to determine whether any difference existed in the distribution of LDMEs among those who incurred MEs in each group.

To adjust for confounding variables between the two groups, we performed a 1:1 propensity score matching using the nearest-neighbor matching method. Propensity scores were estimated using logistic regression models that included all confounding variables described in the Confounding Variables section. Baseline (first-year) MEs and LDMEs were log-transformed [log (expenditure + 1)] to account for their right-skewed distribution. Categorical variables were encoded as dummy variables using one-hot encoding. We chose propensity score matching over covariate adjustment using multiple regression analysis because: (1) the non-linear relationship between covariates and MEs, and (2) the risk of model extrapolation when comparing groups with different covariate distributions.

All statistical analyses were performed using Python (version 3.10.18), with a significance level of less than 5%. The pandas, NumPy, and SciPy libraries were used for data analysis.

## Results

Before propensity score matching, the regular and irregular PDV groups comprised 289 and 392 participants, respectively; after matching, each group included 169 participants. The distribution of propensity scores differed between groups before matching but were comparable after matching (S1 Fig).

Additionally, the standardized differences for all confounding factors were below 0.1, except for employment in the Other division (0.12), the risk of obesity (−0.14), and the risk of being underweight (−0.12; Table 3). The c-statistic was 0.82, indicating that appropriate matching was achieved.

**Table 3:** Participant characteristics.

		Overall (n = 1,891)		Before matching					After matching					
		n or mean	% or SD	Regular PDV group (n = 289)		Irregular PDV group (n = 392)		SMD	Regular PDV group (n = 169)		Irregular PDV group (n = 169)		SMD	*p* values
**Age, years**		41.82	9.38	43.55	8.76	40.29	9.48	0.36	42.38	9.12	43.01	8.64	-0.07	.51
**Sex, n, %**	Male	1,478	78%	188	65%	337	86%	0.51	133	79%	133	79%	0.00	1.00
	Female	413	22%	101	35%	55	14%		36	21%	36	21%		
**Type of division, n, %**	Clerical	633	33%	121	42%	84	21%		50	30%	53	31%		.83
	Sales	390	21%	37	13%	95	24%	-0.29	32	19%	38	22%	-0.09	
	Research	357	19%	68	24%	48	12%	0.30	35	21%	36	21%	-0.01	
	Manufacturing	286	15%	27	9%	104	26%	-0.45	24	14%	22	13%	0.03	
	Other	78	4%	15	5%	23	6%	-0.31	14	8%	9	5%	0.12	
	Technical	147	8%	21	7%	38	10%	-0.03	14	8%	11	7%	0.07	
**Current smoking status, n, %**	Smoker	456	24%	44	15%	134	34%	-0.13	35	21%	41	24%	0.08	.61
	Ex-smoker	305	16%	39	13%	72	18%	0.50	27	16%	22	13%	0.01	
	Non-smoker	1,130	60%	206	71%	186	47%		107	63%	106	63%		
**Exercise frequency, n, %**	Rarely/never	815	43%	114	39%	119	51%	-0.09	74	44%	81	48%	0.05	.87
	1–2 times per month	378	20%	50	17%	81	21%	0.21	33	20%	30	18%	0.03	
	1–2 times per week	554	29%	94	33%	91	23%	0.20	52	31%	50	30%	0.05	
	Over 3 times per week	144	8%	31	11%	21	5%		10	6%	8	5%		
**Recall of dental check-up results, n, %**	YES	1,199	63%	215	74%	219	56%	0.39	110	65%	110	65%	0.00	1.00
	NO	692	37%	74	26%	173	44%		59	35%	59	35%		
**Proportion with risk factors, n, %**	Obesity	473	25%	73	25%	89	23%	0.06	44	26%	55	33%	-0.14	.23
	Underweight	118	6%	24	8%	27	7%	0.05	9	5%	14	8%	-0.12	.39
	High blood pressure	370	20%	58	20%	83	21%	-0.03	39	23%	41	24%	-0.03	.90
	Liver dysfunction	588	31%	90	31%	124	32%	-0.01	53	31%	58	34%	-0.06	.64
	Hyperglycemia	395	21%	69	24%	79	20%	0.09	35	21%	36	21%	-0.01	1.00
	Dyslipidemia	286	15%	37	13%	66	17%	-0.11	25	15%	28	17%	-0.05	.76
**Number of outpatient visits, days**		11.96	12.51	16.16	15.51	8.09	9.77	0.82	11.75	11.29	12.14	11.81	0.02	.83
**MEs, USD**		300	1,087	478	2,043	256	1,015	0.42	334	1,254	418	1,404	-0.00	.32
**LDMEs, USD**		48	226	46	156	52	439	0.20	43	167	103	658	-0.07	.52
**CPI, n, %**	Code 1 (healthy; PPD <4 mm)	1,435	76%	230	80%	286	73%	-0.15	128	76%	132	78%	0.06	.14
	Code 2 or 3 (unhealthy; PPD ≥4 mm)	456	24%	59	20%	106	27%		41	24%	37	22%		

Note: SMD is not calculated for variables marked as blank because they were excluded from the propensity score matching model to avoid multicollinearity.

Abbreviations: CPI, Community Periodontal Index; PPD, probing pocket depth; LDMEs, lifestyle-related diseases medical expenditures; MEs, medical expenditures; SMD, standardized mean difference

Based on post-matching comparison, 65 participants in the regular PDV group and 56 in the irregular PDV group experienced at least one LDME during the 8-year period, with no significant differences in incidence rate. Among participants who incurred LDMEs, the cumulative expenditures from 2016 to 2023 were examined (Fig 2).

**Fig 2:**
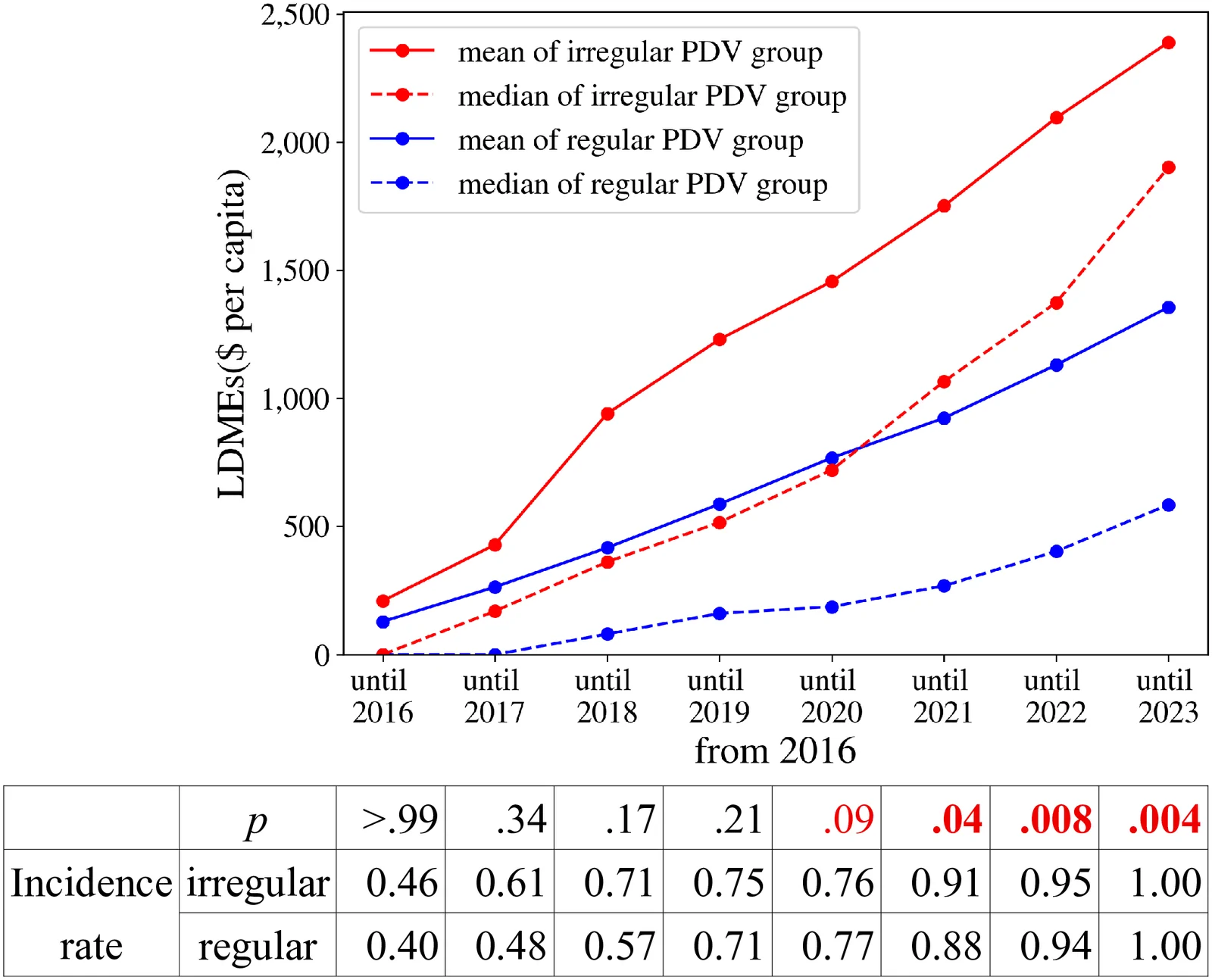
Annual trends in cumulative LDMEs per capita. Note: Following Fisher’s exact test, a one-sided Mann–Whitney U test restricted to individuals with values > USD 0 was conducted. *p*-values in red indicate marginal significance after Bonferroni correction for eight comparisons (α/8): *p*-values in bold red indicate statistical significance after Bonferroni correction for eight comparisons (α/8).

### LDMEs, lifestyle-related disease medical expenditures

Both mean and median values increased over time, showing higher expenditures in the irregular PDV group, although no significant differences were observed during the first 5 years (2016–2020). However, significant differences emerged in the final 3 years (2021 onward) even after adjustment for multiple comparisons. In 2023, the average cumulative 8-year LDMEs in the regular PDV group was USD 1,356 (median USD 585), compared with USD 2,390 (median USD 1,902) in the irregular PDV group. The distribution of cumulative LDMEs at the final year is shown in Fig 3 showing that the regular PDV group had a higher proportion of individuals with lower MEs around USD 0.

**Fig 3:**
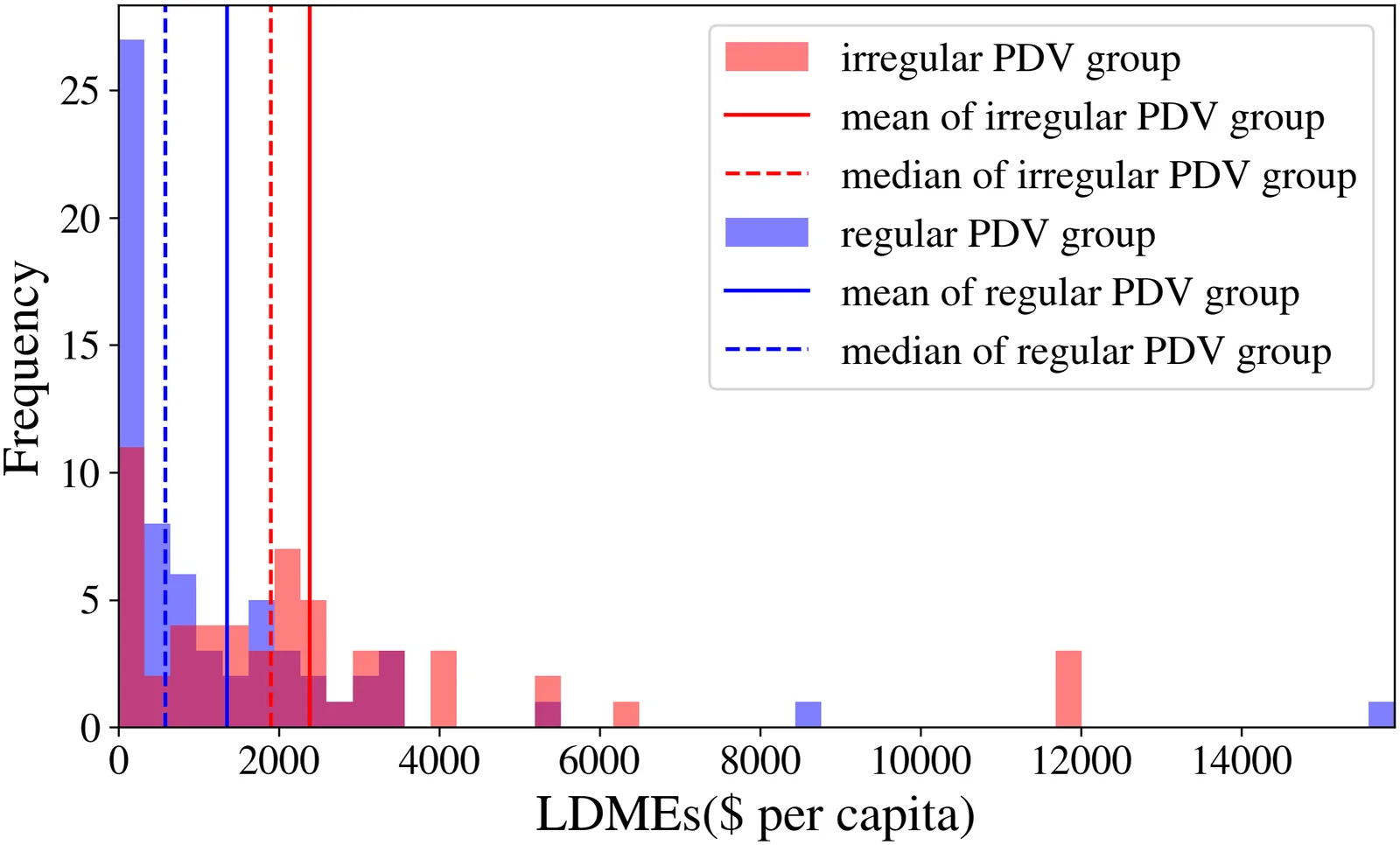
Histogram of cumulative LDMEs per capita over 8 years. LDMEs, lifestyle-related disease medical expenditures; PDVs, preventive dental visits.

In the sensitivity analysis, we changed the threshold for group classification was changed. The regular PDV group was defined as attending PDVs in ≥50% of years and the irregular group as attending PDVs in <50% of years. Propensity score matching yielded 667 participants per group, with standardized differences for all confounders below 0.1 and a c-statistic of 0.70, confirming appropriate matching. Post-matching comparisons showed no significant difference in the proportion of participants incurring any LDME over 8 years (248 and 227 in the regular and irregular PDV groups, respectively). Among those with LDMEs, mean and median cumulative expenditures again increased over time, with widening differences favoring the regular PDV group. Marginally significant differences appeared in the final year (2023), consistent with the primary analysis (Fig 4). In 2023, cumulative 8-year LDMEs averaged approximately USD 1,424 (median USD 752) in the regular PDV group versus USD 1,917 (median USD 1,105) in the irregular group. The final-year distribution is shown in Fig 5.

**Fig 4:**
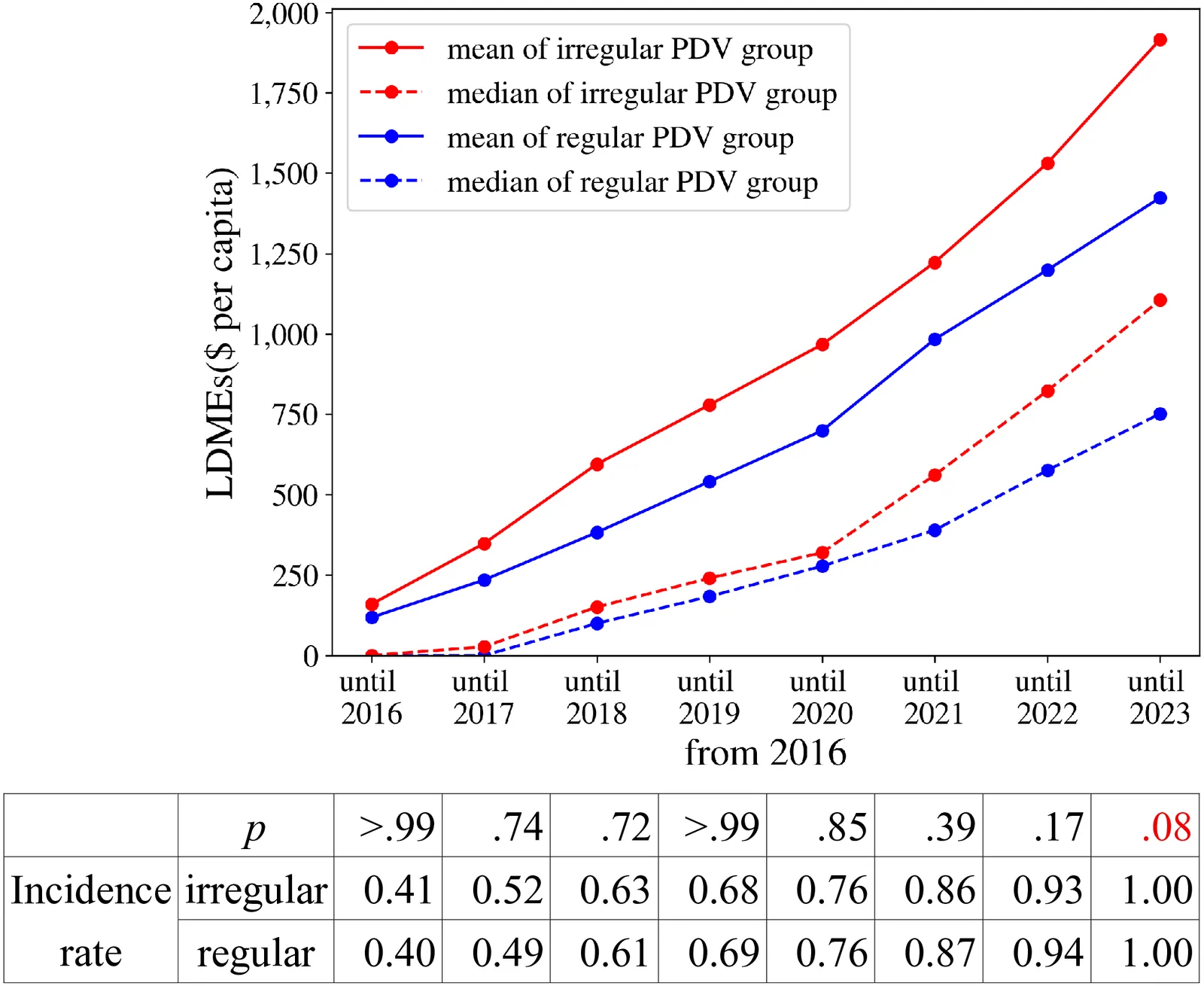
Annual trends in cumulative LDMEs per capita (sensitivity analysis). Note: Following Fisher’s exact test, a one-sided Mann–Whitney U test restricted to individuals with values > USD 0 was conducted; *p*-values in red indicate marginal significance after Bonferroni correction for eight comparisons (α/8). LDMEs, lifestyle-related disease medical expenditures.

**Fig 5:**
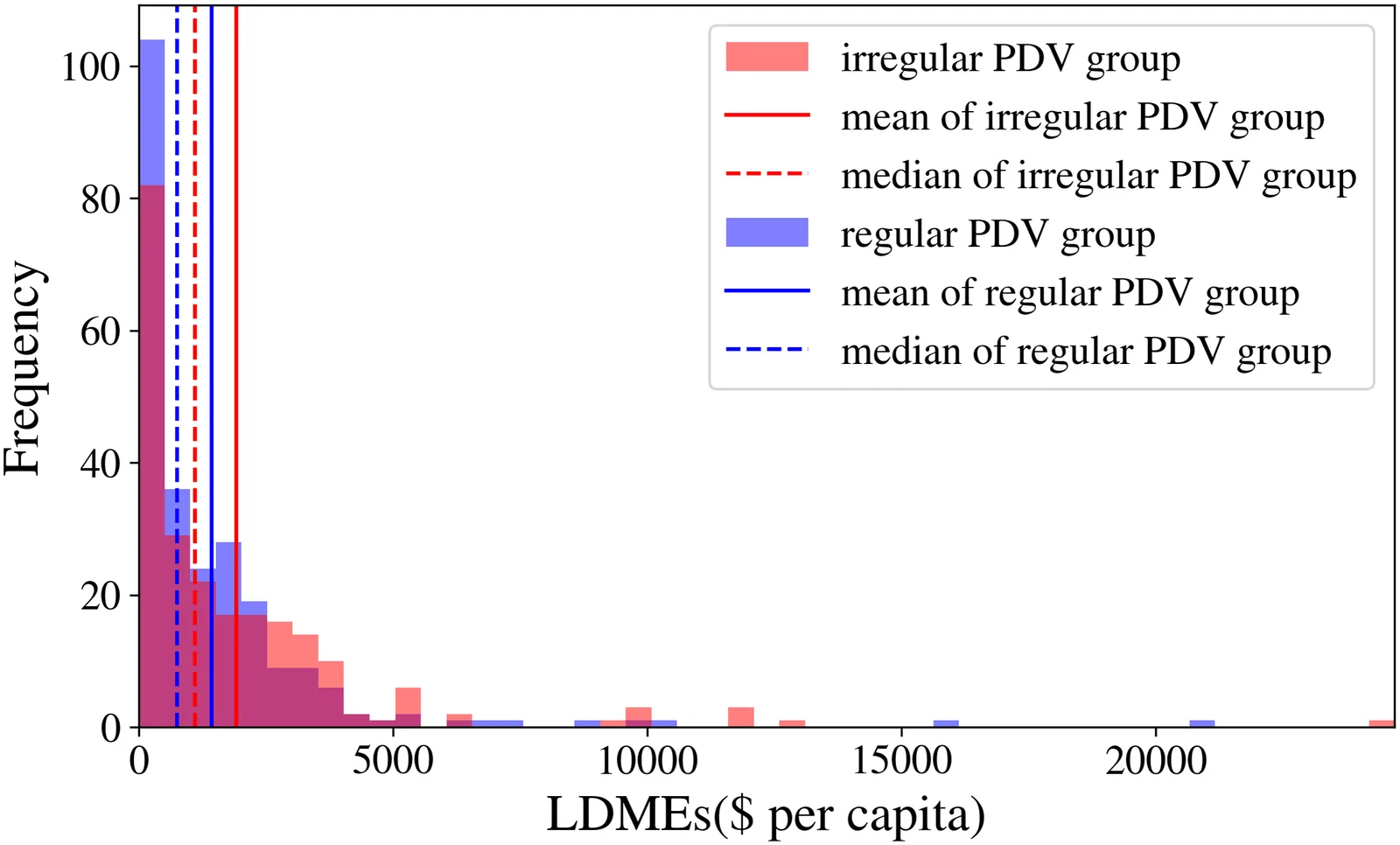
Histogram of cumulative LDMEs per capita over 8 years (sensitivity analysis). LDMEs, lifestyle-related disease medical expenditures; PDVs, preventive dental visits.

We also collected data to examine differences between the groups regarding oral care habits (other than PDVs), oral health status, and overall systemic health (Table 4). Fisher’s exact test was employed for comparisons. In 2023, the regular PDV group exhibited significantly higher floss usage, lower prevalence of periodontal disease and gum bleeding, and a higher proportion of healthy lifestyle habits, compared with irregular PDV group. Notably, these significant differences in 2023 were observed despite adjusting for periodontal disease prevalence and healthy habit proportions as confounding factors at baseline in 2015. Additionally, overall health showed no significant differences although the experience rate was lower in the regular group than in the irregular group. This may partly reflect the 2015 adjustments and the binary categorization of overall health as either “at risk” or “not at risk” with the criteria for risk status defined as shown in S1 Table.

**Table 4:** Comparison of oral health, general health, and lifestyle factors between regular and irregular PDV groups.

Category	Item	Regular PDV group	Irregular PDV group	*p* value
**Oral care habits**	Flossing (≥1/week)	59.2%	31.4%	<0.001
	Brushing teeth at noon	76.9%	68.0%	.088
**Oral health**	Periodontal pocket ≥4 mm	20.1%	33.1%	.010
	Gum bleeding	6.6%	18.0%	.003
**Overall health**	Cumulative 8-year treatment criteria met	43.8%	52.1%	.157
**Lifestyle**	Implementation of lifestyle improvements	39.1%	21.9%	.001
	Current smoking status (Smoker)	8.9%	21.3%	.002
	Exercise frequency (≥1/week)	53.8%	40.8%	.022

Note: Treatment criteria are shown in S1 Table. *p* values were calculated using Fisher’s exact test. Implementation of lifestyle improvements refers to participants who indicated that they had already made changes when asked, “Do you want to improve your lifestyle habits such as exercise and diet?” The proportion of such respondents was calculated.

Abbreviations: PDV, preventive dental visit

## Discussion

### Summary of main findings and Interpretation of results

This study examined the association between regular PDVs and LDMEs among employed adults in Japan using a 9-year historical cohort. While the proportion of participants incurring LDMEs did not differ between groups, among those who did, the irregular PDV group experienced higher LDMEs per capita. Over time, cumulative LDMEs diverged, with the regular PDV group ultimately showing significantly lower costs. This group also had more individuals with minimal expenses (near USD 0) compared with the irregular PDV group.

These findings suggest two important implications. First, since the proportion of participants with lifestyle-related diseases was similar between the groups, differences in MEs is unlikely to reflect avoidance of necessary care in the regular PDV group. Second, the delayed emergence of significant differences (after approximately 5 years) indicates that prolonged observation is necessary to detect the financial impact of PDV attendance.

To our knowledge, this is the first study to investigate the relationship between PDVs and LDMEs.

Three possible mechanisms can be considered for the association between oral care, including PDVs, and LDMEs. First, the improvement of oral health; second, the improvement of general health accompanying better oral health; and third, the suppression of general health deterioration due to increased health literacy.

### Comparison with previous studies

Previous reports showed improvements in oral health through oral interventions [18,19], the impact of oral health on overall health conditions such as metabolic syndrome [20], relationship between oral health and MEs [21], and association between overall health conditions such as obesity and diabetes with MEs [22]. However, the relationship between PDVs and LDMEs had not been reported before. The results of this study reveal that regular PDVs has lower LDMEs, and considering previous findings, it is possible that the maintenance and improvement of oral health through PDVs positively influenced general health, which in turn affected LDMEs as part of the outcome.

Our findings both support and extend previous research examining the relationship between dental care and medical expenditures. Several prior studies have reported associations between preventive dental services and reduced healthcare costs, though with important methodological differences from our study.

Ichihashi et al. [6] compared medical expenditures among three groups categorized by workplace dental check-up frequency (at least once a year, once every 2 years, and less than once every 2 years). Among younger individuals, they observed significant differences only between those visiting once every 2 years or more and those visiting less frequently. Similarly, Lamster et al. [7] reported that among patients with diabetes aged 42–64 years, those receiving preventive dental services had significantly lower non-dental medical expenditures than those who did not receive such services. While these studies provide valuable evidence for the potential economic benefits of dental care, they did not specifically examine which disease categories drive these expenditure differences.

More recently, Taylor et al. [9] investigated the relationship between preventive dental visits and overall medical expenditures using an instrumental variable approach among Medicaid-enrolled adults. However, their study period was limited to analyzing expenditures within a 1-year timeframe, which may have been insufficient to capture the delayed effects of preventive dental care on downstream medical conditions. Given that certain chronic diseases take years to develop, a longer observation period may be necessary to detect the full benefits of regular preventive dental visits.

Our study addresses these gaps by specifically targeting LDMEs to clarify which disease category drives expenditure differences, and by adopting a longer observation period and rigorous confounder adjustment, as detailed below.

### Strengths of this study

This study has four main strengths. First, this study specifically targeted LDMEs rather than overall medical expenditures, providing more precise evidence regarding which disease category is influenced by preventive dental care. Second, it tracks continuous trends in medical, PDV, and receipt data for employed adults over a 9-year period. Observing PDVs across multiple years addresses the limitations of single-year analyses, which may not capture habitual participation and are more susceptible to outliers. Third, in addition to regular PDVs and MEs, we collected detailed information on overall and oral health, as well as health literacy, allowing comparisons between groups while adjusting for confounding factors through propensity score matching. Fourth, the 9-year datasets enabled longitudinal comparisons of LDMEs.

### Limitations

Some limitations should also be noted. The first limitation is related to the data source. Since the study involved employees from a single company, potential biases regarding region, age demographics, and industry sectors could not be excluded. Moreover, since this historical cohort study relies on secondary data not specifically collected for this research, certain variables may be missing or incomplete. For instance, data capture gaps could occur if employees received care outside the company’s health insurance claims system, or if claims data were not fully recorded due to administrative processes. Importantly, PDV attendance was self-reported during in-company dental check-ups, which may not accurately reflect actual participation. Furthermore, LDMEs were determined using the primary diagnosis (“Diagnostic categories code 1”), as complete access to detailed medical records was not available. This approach was adopted because the nature of the data did not allow for cost allocation between primary and secondary diagnoses. It should be noted that this approach may have yielded conservative estimates of LDMEs, as it did not capture cases recorded as secondary diagnoses. Additionally, the exclusion of pharmaceutical expenditures from this analysis should be recognized as an important consideration. However, because this limitation applies equally to both PDV groups, the difference between groups is less likely to be systematically biased, assuming no large differential in data capture.

The second limitation concerns the adjustment for confounding factors using propensity score matching. Although our propensity score matching successfully achieved balance across measured confounders, as indicated by the SMDs reported, the possibility of residual confounding from incompletely measured or unmeasured factors cannot be entirely excluded. However, given the voluntary nature of PDVs and the ethical and practical constraints of conducting a randomized controlled trial, propensity score matching remains a suitable approach for this observational study.

Third, regarding the mechanism by which the association between PDVs and LDMEs arose, this study was limited to comparing LDMEs simultaneously after grouping by PDV. Additional analyses exploring the mechanisms linking PDVs and LDMEs are warranted.

### Future directions

Overall, our findings suggest that PDVs may not only improve oral health but also be associated with moderating LDMEs. These results could inform employer strategies, supporting the implementation or reinforcement of dental check-ups and preventive programs as part of comprehensive employee health initiatives. Future research should strengthen causal inference through time series analyses and further clarify intermediary pathways. In this study, PDVs and LDMEs were observed concurrently (2016–2023). Future studies with longer-term data should separate the observation periods of independent (PDVs) and dependent (LDMEs) variables to better elucidate causal relationships.

## Supporting information

S1 Table Treatment criteria. (DOCX)

S1 Fig Propensity score distributions before and after propensity score matching in the regular and irregular PDV groups. (TIF)

## References

[1] American Medical Association [Internet]. Trends in Health Care Spending. c2025 [cited 15 October 2025] Available from: https://www.ama-assn.org/about/ama-research/trends-health-care-spending

[2] Ministry of Health, Labour and Welfare [Internet]. Future Prospects for Medical and Nursing Care Expenditure. c2018 [cited 15 October 2025] Available from: https://www.mhlw.go.jp/content/12600000/000536591.pdf Japanese.

[3] Neupane S, Florkowski WJ, Dhakal U, Dhakal C. Regional disparities in type 2 diabetes prevalence and associated risk factors in the United States. Diabetes Obes Metab. 2024;26(10):4776–82. doi: 10.1111/dom.15797 39021356

[4] World Health Organization [Internet]. Oral health: Resolution WHA74.5. Seventy-fourth World Health Assembly, Agenda item 13.2. c2021 [cited 27 October 2025]. Available from: https://apps.who.int/gb/ebwha/pdf_files/WHA74/A74_R5-en.pdf

[5] Försäkringskassan [Internet]. Dental care subsidy. c2025 [cited 15 October 2025]. Available from: https://www.forsakringskassan.se/english/dental-care-subsidy

[6] Ichihashi T, Goto A, Myagmar-Ochir E, Haruyama Y, Muto T, Kobashi G. Association between the interval of worksite dental check-ups and dental and medical expenditures: a single-site, 12-year follow-up study in Japan. BMJ Open. 2022;12(10):e063658. doi: 10.1136/bmjopen-2022-063658 36223958 PMC9562720

[7] Lamster IB, Malloy KP, DiMura PM, Cheng B, Wagner VL, Matson JM, et al. Preventive dental care is associated with improved healthcare outcomes and reduced costs for Medicaid members with diabetes. Front Dent Med. 2022;3:952182. doi: 10.3389/fdmed.2022.952182

[8] Takeuchi K, Sato Y, Suma S, Furuta M, Okabe Y, Tanaka T, et al. Associations of oral health status and dental health service utilization with dental and medical expenditures. J Dental Health 2017;67:160–71. doi: 10.5834/jdh.67.3_160

[9] Taylor HL, Holmes AM, Menachemi N, Schleyer T, Sen B, Blackburn J. The Relationship Between Preventive Dental Care and Overall Medical Expenditures. Am J Manag Care. 2024;30:e39–e45. doi: 10.37765/ajmc.2024.894990 PMC11302381 38381547

[10] Grossi SG, Skrepcinski FB, DeCaro T, Zambon JJ, Cummins D, Genco RJ. Response to periodontal therapy in diabetics and smokers. J Periodontol. 1996;67:1094–102. doi: 10.1902/jop.1996.67.10s.1094 8910828

[11] Grossi SG, Skrepcinski FB, DeCaro T, Robertson DC, Ho AW, Dunford RG, et al. Treatment of periodontal disease in diabetics reduces glycated hemoglobin. J Periodontol. 1997;68(8):713–9. doi: 10.1902/jop.1997.68.8.713 9287060

[12] World Health Organization. Oral Health Surveys Basic Methods - 5th edition. Geneva: World Health Organization; 2013 [cited 12 March 2026]. Available from: https://www.who.int/publications/i/item/9789241548649

[13] Nutbeam D. Health Promotion Glossary. Health Promotion International. 1998;13(4):349–64. doi: 10.1093/heapro/13.4.349

[14] Ministry of Health, Labour and Welfare. Survey on Medical Care Benefits: Report for Fiscal Year 2022, Chapter 3 Summary of Survey Results. [cited 6 August 2025]. e-Stat: Portal Site of Official Statistics of Japan. Available from: https://www.e-stat.go.jp/stat-search/files?page=1&layout=datalist&toukei=00450389&tstat=000001044924&cycle=0&tclass1=000001044945&tclass2=000001220980&stat_infid=000040205044&tclass3val=0 Japanese

[15] Japan Health Insurance Association. Basic Information on Insured Persons and Medical Expenses (Fiscal Year 2024). [cited 6 August 2025]. Available from: https://www.kyoukaikenpo.or.jp/g7/cat740/sb7200/sbb7204/r06 Japanese.

[16] Ministry of Health, Labour and Welfare [Internet]. Social Insurance Disease Classification ICD-10. [cited 6 August 2025]. Available from: https://www.mhlw.go.jp/stf/seisakunitsuite/bunya/kenkou_iryou/iryouhoken/database/hokensippei.html Japanese.

[17] Ministry of Health, Labour and Welfare [Internet]. Management of Lifestyle-Related Diseases: Insights from Health Check-up and Medical Claims Data Analysis. c2019 [cited 6 August 2025]. Available from: https://www.mhlw.go.jp/bunya/kenkou/seikatsu/pdf/ikk-j-01.pdf Japanese.

[18] Dahlén G, Lindhe J, Sato K, Hanamura H, Okamoto H. The effect of supragingival plaque control on the subgingival microbiota in subjects with periodontal disease. J Clin Periodontol. 1992;19(10):802–9. doi: 10.1111/j.1600-051x.1992.tb02174.x 1452808

[19] Axelsson P, Lindhe J. Effect of controlled oral hygiene procedures on caries and periodontal disease in adults. J Clin Periodontol. 1978;5(2):133–51. doi: 10.1111/j.1600-051x.1978.tb01914.x 275282

[20] Morita T, Yamazaki Y, Mita A, Takada K, Seto M, Nishinoue N, et al. A cohort study on the association between periodontal disease and the development of metabolic syndrome. J Periodontol. 2010;81(4):512–9. doi: 10.1902/jop.2010.090594 20367094

[21] Albert DA, Sadowsky D, Papapanou P, Conicella ML, Ward A. An examination of periodontal treatment and per member per month (PMPM) medical costs in an insured population. BMC Health Serv Res. 2006;6:103. doi: 10.1186/1472-6963-6-103 16914052 PMC1574303

[22] le Roux CW, Chubb B, Nørtoft E, Borglykke A. Obesity and healthcare resource utilization: results from Clinical Practice Research Database (CPRD). Obes Sci Pract. 2018;4(5):409–16. doi: 10.1002/osp4.291 30338111 PMC6180713

