# Supplementary material for: Role of regular preventive dental visits in moderating lifestyle-related disease medical expenditures among employed adults
Source: PLoS One. 2026 Oct 15;21(10):e0358693. doi: 10.1371/journal.pone.0358693 (PMC13671501; doi:10.1371/journal.pone.0358693)
Supplement: Supplementary file 1 [file pone.0358693.s001.docx]

**Role of regular preventive dental visits in moderating lifestyle-related disease medical expenditures among employed adults**

**Supporting information**

**S1 Fig. Distribution of propensity scores before/after matching.**

**S1 Table. Treatment criteria.**

| **Risk Factor** | **Parameter** | **Treatment Criteria** | **Unit** |
| --- | --- | --- | --- |
| Blood Glucose | Fasting blood sugar | 126 | mg/dL |
|  | HbA1c | 6.5 | % |
| Blood Pressure | Systolic blood pressure | 160 | mmHg |
|  | Diastolic blood pressure | 100 | mmHg |
| Lipids | Triglycerides | 500 | mg/dL |
|  | High-density lipoprotein | 29 | mg/dL |
|  | Low-density lipoprotein | 180 | mg/dL |
| Liver Function | Aspartate aminotransferase | 51 | U/L |
|  | Alanine aminotransferase | 51 | U/L |
|  | Gamma-glutamyl transferase | 101 | U/L |

Note: Participants meeting any one of these criteria are classified as having a risk.
